# Consumption Treated by Gaseous Enemata

**Published:** 1887-07

**Authors:** 


### CONSUMPTION TREATED BY GASEOUS ENEMATA.

The method of treating tuberculosis by gaseous enemata was the invention of Dr. Bergson, of Lyons, France. The method consists in passing carbonic gas through a solution of chloride of sodium and sulphide of sodium. The gas, carefully freed from atmospheric air, is injected by means of a tube with a long nozzle, high up into the rectum. From two to four quarts of the gas are injected once or twice a day, being slowly introduced through a period of fifteen or twenty minutes. Its mode of action is antiseptic, the gas being absorbed and breathed off through the lungs is said to be destructive of the tuberculosis baccilli and exerts a beneficial therapeutic influence upon the diseased lungs. Great results are claimed for this treatment in France, and the method is now being extensively tested in the Northern hospitals. That the patients generally improve under the treatment is everywhere admitted. The cough and expectoration is favorably modified, the night sweats cease, the appetite and general health, in many cases, are wonderfully improved; but whether the method will prove absolutely curative is not yet determined. The apparatus for manufacturing the gas is being put up and sold by several houses, accompanied with descriptions as to the method of preparing and using the gas. One of these may be seen illustrated in the advertisement of E. A. Yarnall, on advertising page 23 of this journal Experiments are now being made with this method in Atlanta; so that we may have something reliable to report from our own observation in a short while upon this new plan of treatment.

